# Our Thanks

**Published:** 1872-02

**Authors:** 


### PART III.

### Editorials, Correspondence and Miscellaneous.

### OUR THANKS.

We sincerely thank those gentlemen who have sent us communications. We will publish as early as possible. We trust our friends everywhere will send us articles.

